# Maryland Clinical Society

**Published:** 1895-03

**Authors:** H. O. Reik

**Affiliations:** 525 N. Howard St., Baltimore, Md.


### MARYLAND CLINICAL SOCIETY.

STATED MEETING HELD JANUARY 18, 1895.

DR. SIMON FLEXNER read a paper on the Pathology and Bacteriology of Diphtheria.

DR. L. F. BARKER then addressed the society upon the Antitoxine treatment of Diphtheria. In part, he said: The antitoxine treatment is a final step in a long series of investigations. The principles underlying it are not new; the methods of immunization now in use are not unlike those used by Pasteur in his earlier work, to which he was undoubtedly stimulated by the success of Jener's vaccination. After it was found out that the symptoms and lesions in the infectious diseases were dependent in the main on the toxic products of bacteria, it was soon discovered that the chemical toxines formed by the bacteria were capable, when introduced in gradually increasing doses into animals, of giving rise to an artificial immunity almost as certainly as the inoculation of the virus itself. Those who were attacking the problems of immunity, that is to say, were endeavoring to discover what changes took place in the body of an individual during and after an infection, such as small-pox which rendered him after thorough recovery, practically insusceptible to a second attack, studied the fluids and tissues of the body, before, during, and after an infection, these investigations led to the formation of two schools: first, that which believes that the normal resistance offered against infection and the immunity acquired by one attack or by artificial means, depend upon certain properties of the blood serum; and, second, that which holds that the activity of the cells of the body accounts for the phenomena of both natural and acquired immunity. Dr. Nuttall showed that the blood serum of an animal that had been immunized against anthrax, when injected into another animal, would kill more anthrax bacilli than the blood serum of a susceptible animal. Other investigators proved that the use of the blood serum from an immunized animal, would, when introduced into another animal, protect it from infection with the same micro-organisms. Then Behring and his assistants demonstrated that the injection of the blood serum of animals rendered artificially immune against diphtheria and tetanus, would heal these infections, even after they were well started in other animals. It was easier to understand how it would be possible to set up an artificial immunity against small-pox or typhoid fever, than against diseases like diphtheria or pneumonia, for the latter are diseases from which an individual may suffer more than once. Immunity has to be looked upon as a relative term and we may speak of a temporary and of a permanent immunity. An animal into which the bacterial toxines are injected, has to suffer a reaction before it becomes immuned, a certain space of time must elapse before the anti-toxines are formed. In the most successful method of producing artificial immunity, one begins by injecting small quantities of the well-diluted poisons. One of the most difficult problems in applying the serum therapy to human beings lay in obtaining a serum which contained the anti-toxic substance in sufficient concentration, so that not too large quantities would have to be used for injection. Larger animals than those usually experimented upon had to be employed and the horse has been for various reasons selected as most suitable. A small dose of diluted diphtheria toxines is at first injected into the region of the shoulder. The animal is somewhat disturbed and does not take its food as usual. After several days a second dose is administered, increasing doses producing less effect, until after a period of from four to six months, the horse is rendered immune and the anti-toxic strength of its serum may have attained a high degree. The serum is tested from time to time as to its anti-toxic power and when sufficient concentration has been reached the blood is drawn, the serum separated, standardized, and enclosed in flasks. Behring's so-called normal serum is of such a strength that one-tenth of one cubic centimetre of it will counteract when injected with it into an animal, ten times the minimum amount of diphtheria poison which is fatal for a guinea-pig weighing three hundred grammes. One cubic centimetre of this normal serum is called an anti-toxine unit. Serum No. 1 of Behring is sixty times as strong as this normal serum, serum No. 2, one hundred times as strong, and serum No. 3, one hundred and forty times as strong. In treating the disease, the earlier the anti-toxine is given, the better will be the result. Of the cases treated during the first two days, practically one hundred per cent. get well. At first too small doses were given, now not less than six hundred units (one flask of No. 1) are given as a beginning dose, and if the case be very severe or be seen late, as much as sixteen hundred units may be given immediately. Within twenty-four hours after the injection, the pulse, as a rule, is slower, the temperature lowered, and the patient feels better in every way. If the cases are not seen until the third or fifth day, when the organs may already be seriously affected, it cannot be expected that the anti-toxine will have such a beneficial effect; it can only counteract the poisons then present; it cannot repair the damage already done. A few relapses have occurred after its use, and some deaths, but these were not, it is claimed, in cases treated from the beginning. Very gratifying statistics come from Germany and France—the mortality rate has been markedly lowered. The disease, Behring states, is now absolutely within the control of the physician. It was thought at first that one-tenth of the ordinary healing dose would suffice to protect those who had been exposed to the disease, from contracting it. But it is now recommended that one hundred and fifty units be injected as a prophylactic or immunizing dose. Some curious after-effects have followed its use, such as urticaria and erythematous eruptions, pains in the joints, sometimes accompanied by swelling, but in no instance were these symptoms of serious import. Laryngeal complications, it is stated, do not develop if the anti-toxine has been used before they appear. It is claimed that tracheotomy is rarely necessary and that intubation will answer in those cases where the larynx is implicated. The anti-toxine is not to be looked upon as a direct chemical antidote, for it does not act against poison in the same manner that an acid neutralizes an alkali; for example, there is evidence in support of the view that the anti-toxine acts indirectly by rendering the cells of the body capable of resisting the action of the toxines. The anti-toxine for one disease may act to some extent in increasing the resistance of the body cells against the toxines of different origin. For instance, while the blood serum of an animal rendered immune against snake poison has no anti-toxic effect against the toxine of tetanus, yet an animal which is immunized against tetanus yields a serum which combats the toxic effect of snake poison, and there are other facts adduced which shake our confidence in the specificity of anti-toxines. There may be, to a certain extent, an over-lapping of the immunities. Diphthera offers, as Buchner has pointed out, a better opportunity for the study of the effects of a new remedy, than does tuberculosis; for while the former approaches more nearly to a typical infection, the latter is almost a typical intoxication. Again while the tuberculosis runs a protracted course as a rule, and is subject to spontaneous exacerbations and ameliorations, diphtheria is an acute process terminating soon either in recovery or in death, and thus is a disease in which conclusions concerning the efficacy or futility of a given method of treatment, may speedily be arrived at. Koch's tuberculin treatment differed from the anti-toxine treatment of diphtheria in that in the former a glycerine extract of cultures of the tubercle bacillus were directly injected into the patient, there to set up a reaction which after a time was to lead to the formation of healing substances, while in the latter, the toxines of the diphtheria bacilli are injected into an animal, the animal suffers the reaction and builds the healing substances, and these are transferred ready-made, to the human being. Should the new treatment of diphtheria prove to be as satisfactory as it promises, the outlook for the cure of infectious diseases in general, is bright. We shall, however, be compelled to wait patiently until the bacteriologists, to whom all the credit of this new treatment is due, have perfected the arrangements for the application of the serum therapy to the other infectious diseases.

DR. N. G. KIERLE explained the difference between the diphtheria in the human, and that of the pigeon and fowls. He exhibited several birds, some having true diphtheria, others the mixed infection.

DR. J. H. BRANHAM reported upon two cases of diphtheria in which he had used the anti-toxine treatment.

CASE 1. Little girl, seven years of age; had been ailing for about two weeks with a slight sore throat and injection of the mucous membrane over the tonsil. The diphtheritic membrane appeared first upon the uvula. At that time the child was not very sick, having a pulse of 90 and temperature 100. He made a small injection of anti-toxine on the 6th; about eighteen hours after the membrane appeared. On the same day a second dose was given, much larger, at about 4 P. M. The pulse was then 120, temperature 101.6. The next morning both pulse and temperature had gone higher, when he changed and gave an injection of a new solution. On the 8th the temperature in the morning was 102, pulse 130. Patient not very much improved. At 9 P. M. a full dose of Behring's solution (12 cubic centimetres) was given. The next morning the temperature after twelve hours, was nearly normal, and the patient proceeded to recover very rapidly. The first solution used was obtained from Pasteur's New York Laboratory, but within twelve hours after giving a full dose of the Behring solution, the patient was very much better and practically has not been sick since.

CASE 2. Patient first seen on the fourth day of disease; had been treated by another physician with the ordinary remedies. Bacteriological examination was made and a dose of the Pasteur material given on the fifth day. On the next day there was a decided manifestation of laryngeal involvement. A full dose of the Behring solution No. 2 was then given. Sixteen hours later, in a fit of coughing, a cast of the larynx was brought up which showed the bacteria. After that time the pulse and temperature came down to normal, and did not rise again. The patient recovered rapidly. A full dose in both cases seemed to act beautifully.

DR. J. F. MARTENET: I desire to report an interesting case in which I had the opportunity to use this remedy. The case was that of a child two years old, who had been sick ten days. It was primarily a laryngeal case. Another physician had been treating it and gave the case up as hopeless. When I saw it the larynx and trachea were full of the membrane and breathing was very difficult. I gave the first injection of anti-toxine that evening, and the second dose the following morning, the respiration having by that time somewhat improved. In the evening, however, it was worse. The larynx was almost occluded and the child could scarcely breathe. The temperature was 103, respiration very rapid; the pulse rapid and weak. I could not get more of the anti-toxine at that time, so I had to try tracheotomy, and left the tube in all night. Next morning the child was apparently dying. We removed the tube, and the child getting more air, improved somewhat. By the next day I had succeeded in obtaining more of the anti-toxine and gave a third injection. Improvement went on rapidly, and by the following day the child was practically well. Bacteriological examination showed it to have been a case of mixed infection. Tracheotomy undoubtedly helped to save the child's life, but it may have been by giving the anti-toxine an opportunity to produce its effect.

*525 N. Howard St., Baltimore, Md.* H. O. REIK, M. D.

---

SULPHATE OF DUBOISINE.—Loiacono and Masuro (*Il Pisani*, XIV) review the literature of duboisine and give their experience in its use in the insane. The following are their conclusions: 1. The neuter sulphate of duboisine of Merck is an excellent sedative in all forms of psychic and motor agitation, especially in the maniacal form. It is superior to the other sedatives used up to the present time in its almost certain prompt effect, in the ease of administration, which is independent of the will of the patient, and in the absence of the inconveniences attending hyoscyamine and hyoscine. 2. It is an excellent hypnotic also, independently of its sedative effect, although in that its action is much more marked. The sleep produced is very similar to physiological slumber. 3. It has no elective action for the female sex and the different results obtained should be attributed to the different degrees of individual reaction. 4. The dose varies from ½ milligramme to 1½ milligrammes. The average dose that, in the great majority of cases, gives all the therapeutic effects without any uncomfortable symptoms, may be stated as about 1 milligramme. The authors also tried the effects of this agent upon a number of epileptics, who under exclusive bromide treatment had very frequent fits, and noted a very marked diminution in the number of the attacks after its use in about two-thirds of the cases or 66.6 per cent. In the remaining 33.3 per cent. it was either not helpful or damaging in its effects. In three cases of status epilepticus it seemed useful in one, and of no advantage in the others. They think as duboisine acts as a sedative, quieting the agitation due to artificial irritation, it will be useful in epilepsy due to the same causes, while it fails in those cases connected with permanent anatomical causes, such as tumors, connective tissue degenerations, etc. While the injections by themselves were largely useful, they found the best effects to follow the combined use of bromides internally with the hypodermic injections.—*Review of Insanity and Nervous Diseases.*

